# Transcriptional Response of ABCH Transporter Genes to Host Allelochemicals in *Dendroctonus armandi* and Their Functional Analysis

**DOI:** 10.3390/insects16111075

**Published:** 2025-10-22

**Authors:** Bin Liu, Jinrui Zhu, Xiaoman Ning

**Affiliations:** Guangxi Key Laboratory of Forest Ecology and Conservation, College of Forestry, Guangxi University, Nanning 530004, China

**Keywords:** *Dendroctonus armandi*, ABCH transporters, plant secondary metabolites, expression patterns, RNA interference

## Abstract

Bark beetles depend on detoxifying enzymes to counteract the defensive terpenoids produced by host trees. Insect ABC transporters play a critical role in the detoxification of insecticides and plant secondary metabolites. However, the specific functions of *ABC* genes in the metabolism of host allelochemicals remain unclear in *D. armandi*. In this study, we observed that verapamil significantly enhanced the mortality of host allelochemicals in beetles, indicating that ABC transporter genes are involved in the metabolism of monoterpenes by *D. armandi*. We then sequenced and characterized the full-length cDNAs of three *ABCH* subfamily genes (*DaABCH1*–*DaABCH3*) from *D. armandi*. Spatiotemporal expression profiling revealed that all three genes were upregulated during developmental transitions (egg to larva and pupa to adult) and tissue-specific enrichment in detoxification-related organs (Malpighian tubules, fat body, and midgut). Additionally, *DaABCH3* expression was detected in the hindgut and brain. Furthermore, *DaABCH1* and *DaABCH2* were significantly induced by treatment with α-pinene and limonene, whereas *DaABCH3* was induced by β-pinene and limonene. Importantly, silencing *DaABCH1* significantly increased mortality in adults fumigated with α-pinene and limonene. These results strongly suggest that *DaABCH1* acts as a key regulator modulating *D. armandi*’s sensitivity to host plant allelochemicals. This finding provides a conceptual basis for developing novel control strategies against this economically significant forest pest.

## Simple Summary

The Chinese white pine beetle (*Dendroctonus armandi*) is a highly destructive forest pest that inflicts significant damage to coniferous ecosystems in the middle Qinling Mountains of China. Insect ATP-binding cassette (ABC) transporters are critically involved in mediating xenobiotic metabolism. However, the roles of *ABC* genes in host terpenoid detoxification remain uncharacterized in *D. armandi*. In the present study, verapamil (an ABC transporter inhibitor) significantly increased beetle mortality induced by four monoterpenes. Then we characterized three *ABCH* subfamily genes. All three genes were upregulated during developmental transitions and predominantly expressed in Malpighian tubules, fat body, and midgut. Notably, *DaABCH1* was significantly induced by α-pinene and limonene and silencing *DaABCH1* significantly increased mortality, which indicated *DaABCH1* represents a critical candidate gene implicated in plant chemical defense mechanisms.

## 1. Introduction

The Chinese white pine beetle, *Dendroctonus armandi*, represents a devastating pest threatening coniferous forests in the central Qinling and Bashan Mountains of China. It not only invades healthy Chinese white pines (*Pinus armandii*) aged 30 years old but also attracts other pest to infest these host trees, thereby disrupting forest ecosystems and causing huge economic losses [1]. Bark beetles spend nearly their entire life cycle beneath the bark of pine trees, except during dispersal to new host trees and the initiation of reproductive activities. A critical phase in their life cycle is host colonization, during which these insects must overcome the tree’s defensive barriers to reproduce successfully [2].

*P. armandii*’s resistance against bark beetles primarily relies on its constitutive and induced physical–chemical defense systems, with constitutive and induced oleoresin terpenes acting as the core defensive components [3]. Oleoresin is a complex mixture composed a lot of monoterpenes, diterpenes, and small quantities of sesquiterpenes [4]. Prior research has identified α-pinene, β-pinene, camphene, myrcene, and limonene as the main components of volatile monoterpenes extracted from *P. armandii* resin [3]. These compounds can harm or kill bark beetles through the toxic properties [5,6]. Although monoterpenes serve as precursors for kairomones or pheromones in the chemical ecology of bark beetles, the insects must metabolize these substances to avoid adverse impacts. Throughout the prolonged coevolutionary arms race between herbivorous insects and plants, insects have evolved sophisticated detoxification enzyme systems. These systems facilitate the breakdown of diverse plant secondary metabolites and synthetic insecticides, thereby endowing insects with remarkable resilience in hostile environments [7].

In insects, xenobiotic detoxification is further divided into three phases, involving multiple detoxification-related enzymes and transporters: cytochrome P450 monooxygenases (P450s) and esterases (ESTs) act as central catalysts in phase I metabolism by mediating oxidation and hydrolysis reactions; glutathione S-transferases (GSTs) operate in phase II via conjugation processes; and ATP-binding cassette (ABC) transporters facilitate phase III through elimination [8]. Studies exploring the tolerance mechanisms of *D. armandi* have shown that elevated activity of detoxifying enzymes (such as P450s, ESTs, and GSTs) is the primary factor contributing to the beetle’s metabolic tolerance to monoterpenes [9,10,11,12]. Although many studies have found that ABC transporters are involved in transporting toxic substances out of cells [13,14,15], it is still unclear whether they participate in the detoxification of monoterpenes in *D. armandi*.

ABC transporter family is one of the largest families of transporter-encoding genes in the metazoans [16]. Most ABC transporters primarily function in the ATP-dependent active transport of a wide range of substrates, such as lipids, peptides, amino acids, polysaccharides and their conjugates, pharmaceuticals, and as a pump that extrudes toxicants from the cells [15,16,17,18]. Moreover, ABC transporters also participate in a broad spectrum of other biochemical and physiological processes, including uric acid uptake, ocular pigmentation, and acting as ion receptors and channels [15,19,20]. Typical ABC transporters contain two highly conserved structural domains: the nucleotide-binding domain (NBD) and the transmembrane domain (TMD) [21]. Between the two domains, NBD binds and hydrolyzes ATP to supply energy for substrate transport, and TMD governs substrate selectivity. Based on the sequence similarity of NBDs, ABC transporters are categorized into eight subfamilies, labeled ABCA to ABCH [16,18].

ABCH represents the sole transporter subfamily absent in humans and other mammals yet conserved across all arthropods, offering an advantageous and biosafe target for novel arthropod-specific pest control agents [16]. It was first discovered in the genome of the fruit fly *Drosophila melanogaster* [22]. ABCH transporters have been linked to lipid transport: RNA interference (RNAi)-mediated knockdown of *ABCH* genes in *D. melanogaster*, *Tribolium castaneum*, *Locusta migratoria*, *Plutella xylostella*, and *Diabrotica virgifera virgifera* leads to high mortality due to desiccation [23,24,25,26,27]. Additionally, ABCH transporters have been found to be differentially induced by xenobiotic exposure in several insect species, including *T. castaneum*, *P. xylostella*, *Anopheles coluzzii*, *Acyrthosiphon pisum*, and *Sitophilus zeamais* [20,28,29,30,31]. However, the role of ABCH transporters in host terpenoid tolerance remains uncharacterized in *D. armandi.*

Since no insecticides have been used against bark beetles at our sampling site, the host tree’s chemical defense is the primary selective pressure on *D. armandi*. Detoxifying enzymes of bark beetles have long been a major research focus for understanding the molecular mechanisms underlying bark beetle host selection and colonization behaviors [32,33,34]. In this study, we first evaluated the synergistic effect of verapamil (an ABC transporter inhibitor) on host allelochemicals. We then cloned and identified three *ABCH* genes from the transcriptome sequencing data of *D. armandi* and constructed a phylogenetic tree using these genes. Furthermore, we analyzed the expression patterns of the three *DaABCH* genes. Additionally, we investigated the functions of these *DaABCH* genes in responding to monoterpenes via RNAi. This study aimed to identify *ABCH* transporter genes in *D. armandi* and elucidate their mechanistic roles in mediating responses to key host monoterpenes, thereby establishing a theoretical framework for targeted pest management strategies.

## 2. Materials and Methods

### 2.1. Insect and Reagent Preparation

Chinese white pine beetles were sampled from *Pinus armandii* trees naturally infested by the beetle at the Huoditang Experimental Forest Station. This station is located on the southern slopes of the central Qinling Mountains, Shaanxi Province, China (33°18′ N, 108°21′ E). The beetles were maintained on a synthetic diet in a laboratory environment controlled at 25 ± 1 °C, 70% relative humidity (RH), and constant darkness [35]. α-Pinene (98%), β-pinene (99%), 3-carene (90%), limonene (95%), and verapamil were purchased from Aladdin Industrial Corporation (Shanghai, China).

### 2.2. Synergism Bioassays

Verapamil (an ABC transporter inhibitor) was used to assess the role of ABC transporters in the tolerance of adult beetles to monoterpenes. 100 μL of verapamil (final concentration of 100 mg⋅L^−1^) was topically applied to the pronotum of individual adult beetles. The acetone used as control groups, respectively. After 24 h, allowing for complete penetration of verapamil into the beetle’s body, survival rates were recorded. The adults were then fumigated with different monoterpenes (final concentration of α-pinene, β-pinene, 3-carene and limonene is 36 μL⋅L^−1^, 43 μL⋅L^−1^, 15 μL⋅L^−1^ and 10 μL⋅L^−1^) for 24 h. Following fumigation, the beetles were transferred to an artificial climate cabinet, and mortality was observed at 48 h post-fumigation. Each treatment consisted of three biological replicates, each of which used 40 males and 40 females.

### 2.3. Cloning and RACE

Total RNA was extracted from three developmental stages of *D. armandi* (larvae, pupae, and adults of both sexes), and RNA concentration and quality were determined as previously described [35]. Partial sequences of three *DaABCH* genes were retrieved from the *D. armandi* transcriptome database [10] and used for primer design (Table S1). Full-length cDNA sequences of *DaABCH* genes were obtained through 5′ and 3′ RACE and verified as reported previously [36]. Briefly, cDNA-specific primers for 5′ and 3′ RACE (Table S1) were designed based on the partial sequences. RACE-ready cDNA was synthesized from total RNA using the SMARTer RACE cDNA Amplification Kit (Clontech Laboratories Inc., Mountain View, CA, USA) in accordance with the manufacturer’s protocol.

### 2.4. Bioinformatic Analysis

The three cDNA sequences have been submitted to GenBank under the accession numbers provided in Table 1. Open reading frames (ORFs) of the full-length cDNAs were identified by ORF Finder (https://www.ncbi.nlm.nih.gov/orffinder/, accessed on 16 July 2025). Multiple sequence alignments were carried out using DNAMAN 6.0. The molecular weights (MW) and isoelectric points (pI) of the deduced proteins were predicted using ProtParam (http://web.expasy.org/protparam/, accessed on 16 July 2025). The secondary structures of deduced *DaABCHs* amino acid sequences were predicted using the NPS-SOPMA tool [37]. Phylogenetic trees were built using the neighbor-joining method in MEGA 6.0 [38].

### 2.5. Insect Sampling and Treatments for RT-qPCR

Eggs of *D. armandi* were classified into two developmental substages: early-stage eggs (exhibiting light coloration) and late-stage eggs (proximal to eclosion). Larvae were categorized into two distinct phases: feeding larvae (actively consuming host phloem for growth) and mature larvae (having terminated feeding activity). Pupal development was segmented into early pupae (recently metamorphosed from larvae) and late pupae (approaching adult morphogenesis). Adults were further differentiated into three subgroups: teneral adults (characterized by light cuticular pigmentation), emerged adults, and attacking adults (engaged in colonization of new host trees). Six individuals were pooled as one sample. Antennae, brain, foregut, midgut, hindgut, fat body, Malpighian tubules, reproductive organs, and hemolymph of emerged adults were dissected, ten individuals were pooled as one sample. Subsequently frozen in liquid nitrogen and maintained at −80 °C for long-term preservation until further analysis.

Monoterpenes stimulation was performed using the same method as that in the fumigant toxicity assay. Each treatment group contained 40 males and 40 females of roughly the same size. After 12, 24, and 36 h of exposure to different monoterpenes, beetles surviving in each group were euthanized using liquid nitrogen. An additional 15 individuals per sex were collected at the initial time point (0 h) as controls. All samples were subsequently utilized for real-time PCR analysis, with three biological replicates performed per treatment.

The β-actin gene of *D. armandi* (accession number: KJ507199.1) was used as the reference gene for qRT-PCR [10]. Gene-specific primers were employed to detect the expression of the three *DaABCH* genes (Table S1). qRT-PCR was performed as described in our previous study [35]. All treatments were performed in three technical replicates, and relative gene expression was quantified via the 2^−ΔΔCt^ method [39].

### 2.6. Double-Strand RNA (dsRNA) Synthesis and Injection

The T7 Ribo-MAX™ Express RNAi System (Promega, Madison, WI, USA) was used for dsRNA synthesis [36]. RNA interference primers (Table S1) were designed using the complete *DaABCH* gene sequences. The resulting double-stranded RNA (dsRNA) products were diluted to a concentration of 1000 ng/μL using DEPC-treated water, preserved at −80 °C, and utilized within a six-month period. Newly emerged adult *D. armandi* beetles were microinjected with 0.15 μL of dsRNA solution employing a Hamilton Micro-liter™ syringe (700 series) fitted with a 32 G fine-point needle (Hamilton, Bonaduz, Switzerland). Injection of dsGFP served as the control treatment. Following injection, the beetles were maintained in an environmentally controlled chamber under standardized conditions. Each experimental group included three biological replicates (with independent microinjection procedures), with 60 beetles of each sex injected per biological replicate. Survival was recorded at 48 h post-injection. Six randomly selected adults from each treatment were collected at 24, 48, and 72 h post-injection for qRT-PCR analysis. At 48 h post-injection, adults of both sexes were treated with distinct monoterpenes following established protocols, maintained under controlled conditions for 48 h, and mortality rates were recorded. All treatments were performed with three biological replicates.

### 2.7. Statistical Analysis

All statistical analyses were performed using SPSS Statistics v.25.0 (IBM, Chicago, IL, USA). One-way analysis of variance (ANOVA) followed by Tukey’s post hoc test was used to verify the significance of differences among multiple groups. Student’s *t*-test was applied for two-sample comparisons (* *p* < 0.05, ** *p* < 0.01, *** *p* < 0.001). The results were reported as mean ± standard error (SE). Graphs were generated using Prism 6.0 (GraphPad Software, San Diego, CA, USA).

## 3. Results

### 3.1. Synergistic Effect of Verapamil on Monoterpenes

**Figure 1:**
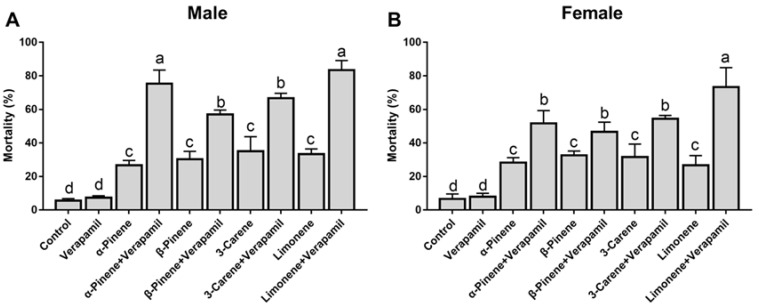
The synergistic effects of verapamil on the toxicity of four terpenoids to *Dendroctonus armandi* male (**A**) and female (**B**) adults. Different lowercase letters indicate significant differences at *p* < 0.05. Post hoc Tukey tests following one-way analysis of variance (ANOVA). All values are mean ± SE; n = 3 biological replicates.

Treatment of *D. armandi* adults with verapamil for 48 h had no impact on mortality (Figure 1), indicating that the inhibitor itself did not impair the beetles’ physiological condition. However, verapamil treatment led to a significant rise in adult mortality following exposure to α-pinene, β-pinene, 3-carene, and limonene: mortality increased by 48.7%, 26.2%, 31.6%, and 50.1% in males (Figure 1A, *p* = 0.032) and by 23.3%, 14.0%, 22.7%, and 46.6% in females, respectively (Figure 1B, *p* = 0.021).

### 3.2. Sequencing and Bioinformatic Analysis

Based on the *D. armandi* transcriptome database, three putative *ABCH* genes (named *DaABCH1*–*DaABCH3*) with complete ORFs were identified. The deduced amino acid sequences of these genes ranged from 710 to 777 amino acids in length (Table 1). The predicted molecular weights varied from 79.36 to 85.94 kDa, and the isoelectric points ranged from 6.12 to 7.81 (Table 1). Signal peptide prediction showed that none of the encoded proteins contained a signal peptide, confirming they are non-secretory proteins. SMART analysis revealed that all *DaABCH* proteins possess transmembrane regions, and transmembrane helix prediction indicated that each *DaABCH* protein has 5–7 transmembrane helices—consistent with their classification as membrane proteins (Table 1). Moreover, all *DaABCH* proteins contain potential N-glycosylation sites, O-glycosylation sites, and phosphorylation sites. In addition, secondary structure analysis showed that all *DaABCH* proteins are mainly composed of α-helices (39.57–40.82%), with a small proportion of β-turns (5.20–6.56%; Table 1).

**Figure 2:**
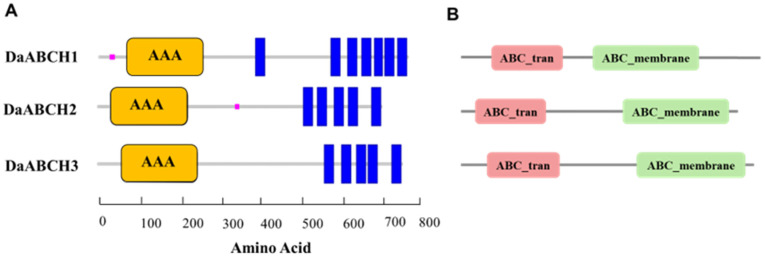
Conserved domain analysis of *D. armandi* ABCH transporters. (**A**) The yellow regions containing AAA modules indicate nucleotide-binding domains (NBDs). The AAA signature signifies ATPases associated with diverse cellular functions. Blue rectangular areas correspond to transmembrane domains, while purple highlighted segments denote low-complexity regions. (**B**) The ABC_tran represents NBD. The ABC2_membrane represents TMD.

All *DaABCH* genes consist of AAA domains (Figure 2A). Moreover, each *DaABCH* gene encodes a single ABC_tran structural domain (NBD) and one ABC2_membrane domain (TMD; Figure 2B). Thus, all *DaABCH* transporters exhibit a reverse domain arrangement (NBD-TMD) and are classified as half-transporters (HTs).

**Figure 3:**
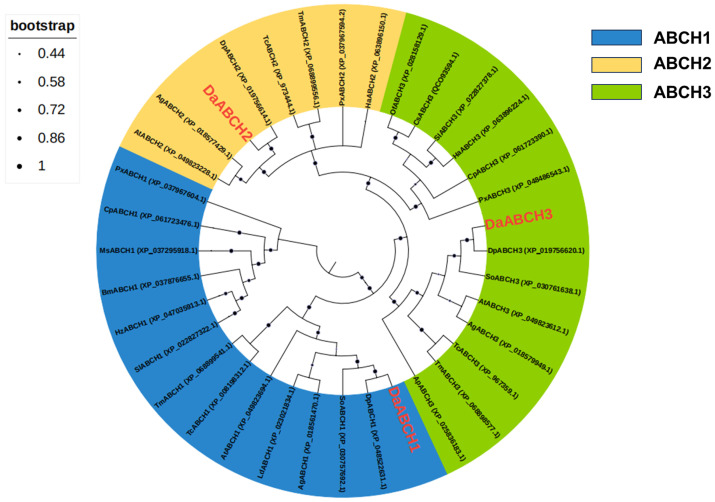
Phylogenetic analysis of *D. armandi* ABCH transporters with other insect species. The phylogenetic tree constructed in MEGA 6.0 using the neighbor-joining method. Bootstrap values (1000 replicates) are indicated next to the branches and GenBank accession numbers are shown behind the insect species. ABCH amino acid sequences are from *D. armandi* (Da), *D. ponderosae* (Dp), *Tribolium castaneum* (Tc), *Sitophilus oryzae* (So), *Aethina tumida* (At), *Anoplophora glabripennis* (Ag), *Tenebrio molitor* (Tm), *Leptinotarsa decemlineata* (Ld), *Plutella xylostella* (Px), *Cydia pomonella* (Cp), *Chilo suppressalis* (Cs), *Ostrinia furnacalis* (Of), *Helicoverpa armigera* (Ha), *Manduca sexta* (Ms), *Spodoptera litura* (Sl), *Helicoverpa zea* (Hz), and *Bombyx mori* (Bm). The *D. armandi* ABCHs are highlighted with red colors.

Phylogenetic trees constructed using *ABCH* subfamily genes from *D. armandi* and *T. castaneum* showed a one-to-one orthologous relationship between *DaABCH* and *TcABCH* genes. This suggests that the number and function of *ABCH* genes are relatively conserved in coleopteran beetles (Figure 3). In addition, the amino acid sequence similarity between *DaABCH* and *TcABCH* proteins ranged from 59.6% to 82.3%, indicating that the amino acid sequence of ABCH transporters is highly conserved.

### 3.3. Expression Profiles Across D. armandi Developmental Stages and Tissues

**Figure 4:**
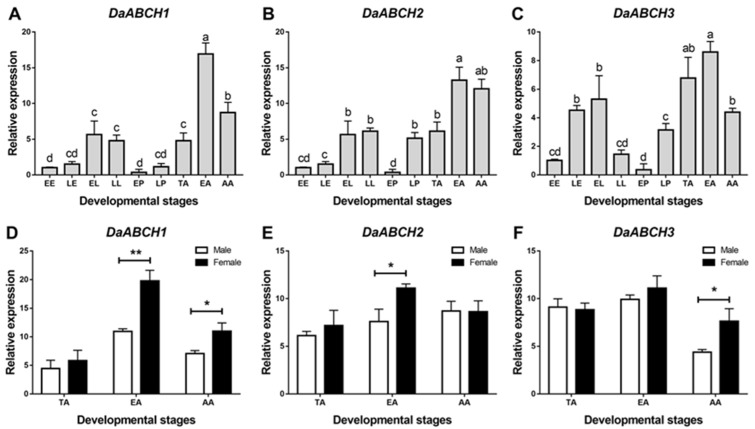
Relative mRNA expression levels of *DaABCH1* (**A**,**D**), *DaABCH2* (**B**,**E**) and *DaABCH3* (**C**,**F**) in different developmental stages and adults of *D. armandi*. Different lowercase letters indicate significant differences at the 0.05 level. The asterisk indicates a significant difference between female and male expression levels (* *p* < 0.05, ** *p* < 0.01, independent Student’s *t*-test). EE, early egg; LE, late egg; EL, early larvae; LL, late larvae; EP, early pupae; LP, late puage; TA, teneral adult; EA, emerged adult; AA, attacking adult. All values are mean ± SE; n = 3 biological replicates.

The three *DaABCH* genes were widely expressed across all developmental stages of *D. armandi*, though with distinct patterns: all were upregulated during developmental transitions (from egg to larva and from pupa to adult; Figure 4). The expression level was highest in emerged adults and lowest in early pupae (*DaABCH1*: *p* = 0.015; *DaABCH2*: *p* = 0.024; *DaABCH3*: *p* = 0.017) (Figure 4). Furthermore, the expression level of *DaABCH1* and *DaABCH2* was significantly higher in females than in males in the emerged adult stage, while *DaABCH3* showed higher expression in females than in males in the attacking adult stage (Figure 4F, *p* = 0.035).

**Figure 5:**
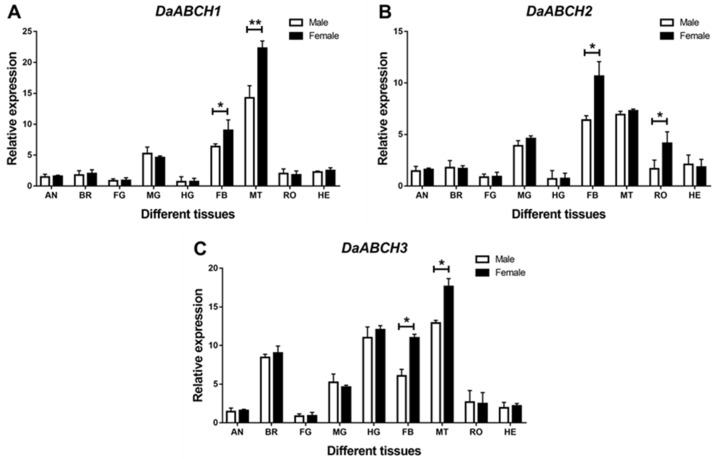
Relative expression levels of emerged adults of *DaABCH1* (**A**), *DaABCH2* (**B**) and *DaABCH3* (**C**) in different tissues of *D. armandi*. The asterisk indicates a significant difference between female and male expression levels (* *p* < 0.05, ** *p* < 0.01, independent-sample *t*-test). All values are mean ± SE; n = 3 biological replicates.

The *DaABCH* genes displayed tissue-specific expression patterns, with sex differences observed in some tissues (*DaABCH1*: *p* = 0.023; *DaABCH2*: *p* = 0.009; *DaABCH3*: *p* = 0.004) (Figure 5). Specifically, *DaABCH1* and *DaABCH2* were highly expressed in the midgut, fat body, and Malpighian tubules, with minimal expression detected in other tissues (Figure 5). *DaABCH3* was mainly expressed in the Malpighian tubules, followed by the hindgut and brain (Figure 5C). Additionally, *DaABCH1* and *DaABCH3* showed higher expression in females than in males in the fat body and Malpighian tubules (Figure 5), while *DaABCH2* exhibited higher female-biased expression in the fat body and reproductive organs (Figure 5B).

### 3.4. Exposure to Monoterpenes

**Figure 6:**
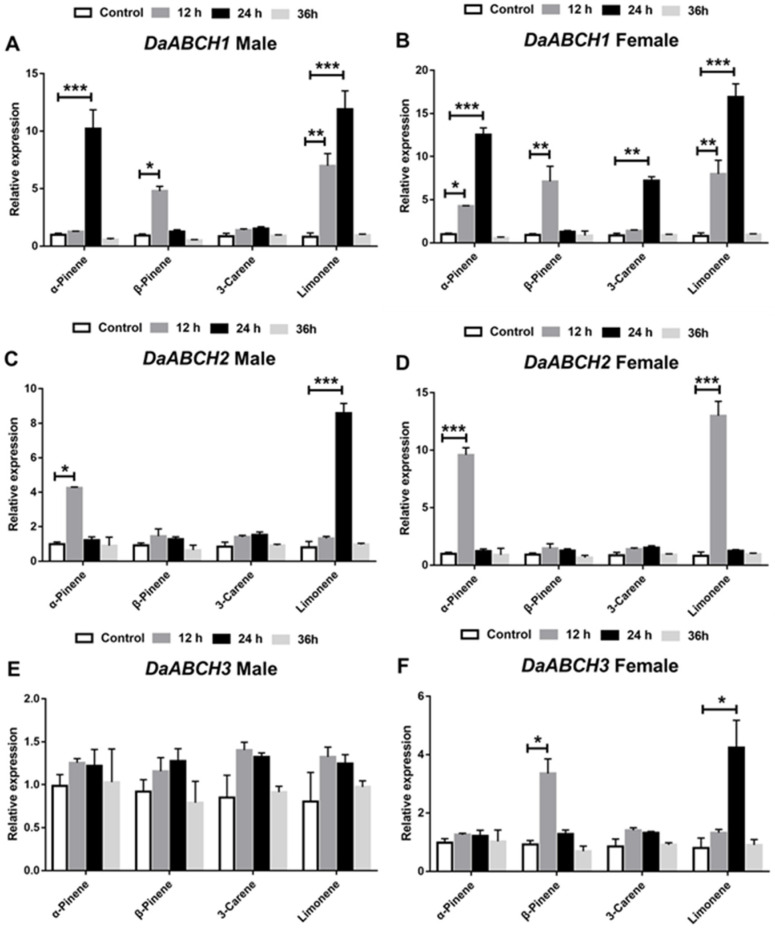
Relative expression levels of *DaABCH1* (**A**), *DaABCH2* (**C**), *DaABCH3* (**E**) in emerged male adults and *DaABCH1* (**B**), *DaABCH2* (**D**), *DaABCH3* (**F**) in emerged female adults of *D. armandi* after stimulation with four terpenoids at exposure times of 12, 24 and 36 h. The asterisk indicates a significant difference between control and treatment groups (* *p* < 0.05, ** *p* < 0.01, *** *p* < 0.001, independent-sample *t*-test). All values are mean ± SE; n = 3 biological replicates.

As shown in Figure 6, monoterpenes treatment induced the expression of the three *DaABCH* genes to varying degrees in *D. armandi* adults, except for *DaABCH3* in males (Figure 6E, *p* = 0.26). In female adults, the relative expression of *DaABCH1* was significantly increased following terpenoid exposure at all time points (Figure 6B). Notably, *DaABCH1* and *DaABCH2* were strongly induced by α-pinene and limonene, while *DaABCH3* was induced by β-pinene and limonene.

### 3.5. Functional Analysis of DaABCH Genes by RNAi Silencing

**Figure 7:**
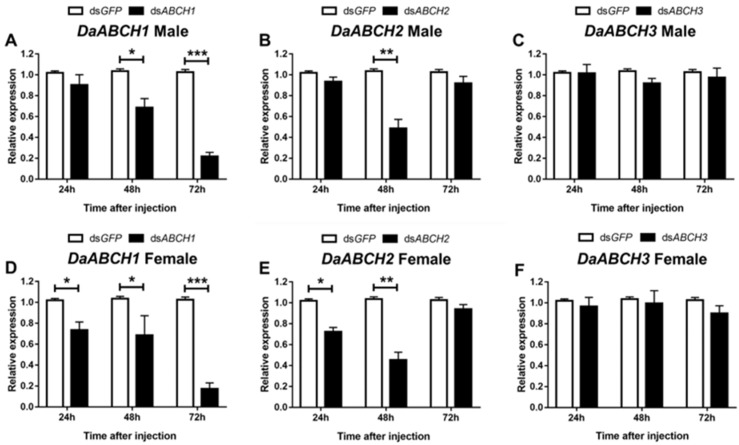
Relative expression levels of *DaABCH1* (**A**), *DaABCH2* (**B**), *DaABCH3* (**C**) in emerged male adults and *DaABCH1* (**D**), *DaABCH2* (**E**), *DaABCH3* (**F**) in emerged female adults of *D. armandi* after injected dsRNA at different time points (24, 48 and 72 h). The asterisk indicates a significant difference between control and treatment groups (* *p* < 0.05, ** *p* < 0.01, *** *p* < 0.001, independent-sample *t*-test). All values are mean ± SE; n = 3 biological replicates.

To further explore the role of *DaABCH* genes in host chemical defense, we suppressed the expression of these genes via RNAi in adults. Compared with the control group, *DaABCH1* expression was significantly downregulated at 24, 48, and 72 h post-dsRNA injection in emerged adults, except at 24 h in males (Figure 7A, *p* = 0.072). *DaABCH2* expression was significantly downregulated at 48 h post-RNAi, while *DaABCH3* expression showed no significant reduction (Figure 7C,F). The maximum reduction in *DaABCH1* expression reached 78.3% (males, *p* < 0.001) and 55.6% (females, *p* < 0.001), while the maximum reduction in *DaABCH2* expression was 79.5% (males, *p* = 0.003) and 58.4% (females, *p* = 0.004, Figure 7).

No statistically significant differences in survival rates were detected in adults injected with dsGFP (control) and those injected with ds*DaABCH1* or ds*DaABCH2* at 48 h post-injection (Figure S1; Male: *p* = 0.15; Female: *p* = 0.32), confirming that the injection procedure itself did not affect the beetles’ physiology.

**Figure 8:**
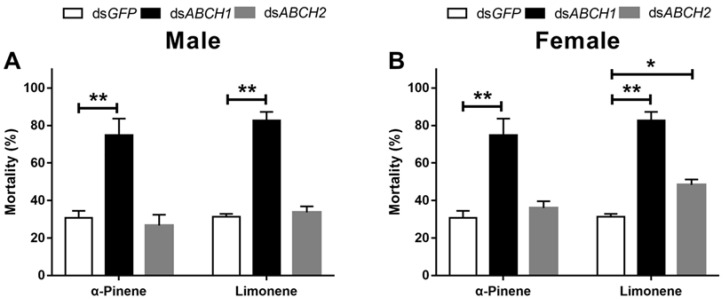
The mortality of *D. armandi* emerged male (**A**) and female (**B**) adults exposed to terpenoids at 48 h after dsRNA injection. The asterisk indicates a significant difference between treatment and control groups (* *p* < 0.05, ** *p* < 0.01, independent-sample *t*-test). All values are mean ± SE; n = 3 biological replicates.

Adults injected with ds*DaABCH1* showed significantly higher mortality than controls after exposure to α-pinene and limonene (Figure 8A,B). Specifically, mortality increased by 46.8% and 52.1% in males, and by 44.9% and 49.2% in females, at 48 h post-exposure to α-pinene and limonene, respectively (Figure 8). In contrast, silencing *DaABCH2* significantly increased mortality only after limonene exposure in female adults (Figure 8B).

## 4. Discussion

The role of ABC transporters in insecticide detoxification has been extensively studied using ABC transporter inhibitors. Evidence supporting the transport function of ABC transporters typically comes from the quantification of transcript or protein levels and synergism assays using ABC inhibitors. For example, in *Apis mellifera*, verapamil treatment significantly increased bee mortality after insecticide exposure [40]. In *T. castaneum*, verapamil inhibited the elimination of the fluorochrome Texas Red from fat body cells [41]. Verapamil has also been shown to enhance the toxicity of diflubenzuron and temephos against fourth-instar larvae of *Aedes caspius* [42], and to increase the toxicity of chlorantraniliprole and abamectin in *Chilo suppressalis* [43,44]. Additionally, the combination of verapamil and tannic acid reduced the survival rate of *A. pisum* compared with tannic acid treatment alone [20]. In this study, verapamil treatment significantly increased *D. armandi* mortality following exposure to host allelochemicals, suggesting that ABC transporters are also involved in the detoxification of monoterpenes in *D. armandi*.

Bioinformatic analyses provided insights into the potential functions of *DaABCH* proteins. Signal peptide prediction confirmed that *DaABCH* proteins are non-secretory, while transmembrane helix prediction identified them as membrane proteins with 5–7 transmembrane helices. The presence of predicted phosphorylation and glycosylation sites indicates that *DaABCH* proteins undergo post-translational modifications, which may be involved in cell signal transduction process. Phylogenetic analysis of the ABCH subfamily revealed a clear orthologous relationship between *DaABCH* and *TcABCH* genes, indicating functional conservation across coleopteran species.

Most insect species have only three ABCH members; however, 8–15 ABCH homologs have been identified in certain hemipteran, including *Bemisia tabaci*, *Lygus hesperus*, and *Diaphorina citri* [45,46,47]. This expanded gene set likely originated from lineage-specific duplication events. In contrast, *D. armandi* possesses a limited ABCH transporter repertoire, comprising only three members, suggesting potential evolutionary or functional specialization. ABCH transporters were first annotated in *D. melanogaster* [22], and subsequent studies have identified *ABCH* genes in various metazoans [16], such as the nematode *Caenorhabditis elegans*, the sea urchin *Strongylocentrotus purpuratus*, the green spotted pufferfish *Tetraodon nigroviridis*, and the zebrafish *Danio rerio* [48,49,50,51].

Previous studies have reported that *ABCH* genes are upregulated with age in some insect species [20,52,53]. RT-qPCR analysis of *DaABCH* expression across developmental stages showed that these genes are expressed throughout the *D. armandi* life cycle, with the highest transcript levels in larvae and adults, and the lowest in pupae. This pattern may be associated with xenobiotic detoxification during feeding (larval and adult stages). These results suggest that *DaABCH* genes play a stage-specific role. Stage-specific expression of *ABCH* genes has been linked to physiological function in *Tribolium castaneum* and *Locusta migratoria* [24,26]. For example, the transcription level of *LmABCH-9C* in *L. migratoria* peaks after molting; knock-down of *ABCH-9C* reduces cuticular lipids in both *L. migratoria* and *T. castaneum*, leading to desiccation and death. The stage-specific expression patterns of *ABCH* genes support their potential functional specialization, though further validation is required in *D. armandi*.

Tissue-specific expression of *ABCH* genes in insects often reflects their physiological roles. Several tissues, including the fat body, Malpighian tubules, and midgut, are known to be involved in insecticide detoxification across different insect species. For instance, the insect fat body contributes to innate immunity and detoxification [54], while the Malpighian tubules play a key role in the detoxification of xenobiotic [55]. In this study, the three *DaABCH* genes showed high transcript levels in the Malpighian tubules, fat body, and midgut, a pattern consistent with observations in *T. castaneum* and *Chrysomela populi* [30,56]. This suggests that *DaABCH* genes are involved in xenobiotic detoxification. In contrast, *BdABCH* genes in *Bactrocera dorsalis* showed relatively low expression in detoxification-related tissues [53]. The differential expression patterns of *ABCH* genes across insect species highlight their functional versatility in fundamental physiological processes. Furthermore, *DaABCH3* exhibited high expression in the hindgut, which is consistent with its expression profiles in *B. dorsalis* and *L. migratoria* [26,53]. Previous studies have shown that hindgut osmoregulatory capacity is associated with cold tolerance in *B. dorsalis* and *L. migratoria* [53,57], suggesting that *DaABCH3* may be involved in hindgut reabsorption in cold-acclimated *D. armandi*. Meanwhile *DaABCH2* showed a higher expression in reproductive organs of the female adult, which may be related to the reproduction of *D. armandi*. These hypotheses require further verification.

Previous reports have indicated that genes upregulated by xenobiotic exposure are generally involved in xenobiotic detoxification. For example, the relative expression levels of *ABCH3*, *ABCH4*, *ABCH8*, and *ABCH10* in *Aphis craccivora* were significantly upregulated in response to imidacloprid treatment [52]. In *A. pisum*, *ApABCH2* expression was upregulated after tannic acid exposure [20]. Additionally, *ABCH* genes were strongly upregulated in *S. zeamais* exposed to terpinen-4-ol [29], and in larvae of *Monochamus alternatus*, ABC transporters exhibit specific responsiveness to oleoresin terpenoids derived from *Pinus massoniana* [58]. Monoterpenes are volatile; when damaged phloem exudes resin, these compounds coat the surface of mining beetles and may enter the beetle’s body via the respiratory system, cuticle, or digestive tract. This study focused on respiratory exposure to monoterpenes. Our results showed that only *DaABCH1* expression was significantly increased after almost four monoterpenes exposure, indicating that this gene plays the most critical role in the detoxification of monoterpenes in *D. armandi*. Moreover, the expression levels of *DaABCHs* in female adults exhibited greater variability compared to males following monoterpene exposure, potentially attributable to female adults enter the bark earlier than males, and they were more sensitive to host volatiles and more important in the location and detoxification of host monoterpenes than males. Although the current results provide important evidence of the role of *DaABCH1* genes in bark beetles’ adaptation to host chemical defense mechanisms, the functions of *ABC* genes from other subfamilies remain undetermined. Moreover, previous studies have demonstrated that other detoxification systems also play key roles in host adaptation [9,10,11,12], which suggests potential functional crosstalk among these detoxification enzymes. Further research should focus on all other *ABC* genes to elucidate their network-level defense mechanisms.

RNAi is a valuable tool for investigating the physiological functions of insect *ABCH* genes in xenobiotic detoxification [16,31,52]. To examine the potential roles of DaABCH, we silenced these genes. The results showed effective downregulation of *DaABCH1* and *DaABCH2* genes, though with differences in silencing efficiency and duration. Notably, silencing *DaABCH1* significantly increased adult mortality following exposure to α-pinene and limonene, identifying *DaABCH1* as a key candidate gene involved in host chemical defense. α-Pinene is the most abundant volatile monoterpene in *P. armandii* resin [3], while limonene causes higher beetle mortality in fumigant toxicity assays than other terpenoids [10]. This finding is consistent with prior studies on *Aphis craccivora*, where dsRNA feeding suppressed *ABCH3* and *ABCH4* expression, increasing *A. craccivora* sensitivity to imidacloprid [52]. Similarly, dsRNA-mediated silencing reduced *ABCH2* transcript levels by approximately 80% in whole female mosquitoes, significantly increasing pyrethroid toxicity [31]. Furthermore, ^14^C-deltamethrin penetration assays showed that *Anopheles coluzzii* lacking this transporter exhibited increased penetration of the radiolabeled insecticide after brief contact [31]. Additionally, RNAi *PxABCH1* can cause 100% larval and pupal mortality in a Bt Cry1Ac-resistant strain of *P. xylostella* [28]. In contrast, silencing *ABCH* genes in *T. castaneum* did not significantly increase mortality following exposure to the pyrethroid cyfluthrin or the diacylhydrazine tebufenozide [30], and while *ApABCH2* was upregulated in *A. pisum* exposed to tannic acid, silencing *ApABCH2* had no effect on tannic acid-induced mortality [20]. These results emphasize that increased transcript levels in response to xenobiotic exposure do not always indicate a role in detoxification, they may instead reflect a non-specific stress response.

However, the RNAi silencing achieved only partial knockdown, leaving room for residual gene activity and potential compensation by other detoxification pathways, and off-target effects were not rigorously excluded. The present results remain inferential in this respect as long as CRISPR/Cas knock-out experiments have not been carried out. Future work will involve conducting direct transport assays combined with CRISPR/Cas9 knockout technology to provide definitive evidence of the role of DaABCH proteins in terpenoid export or sequestration.

## 5. Conclusions

In summary, this study provides preliminary information on the gene structures, sequence characteristics and expression patterns of three *ABCH* genes in *D. armandi*. Verapamil treatment significantly increased *D. armandi*’s susceptibility to host monoterpenes. Additionally, silencing *DaABCH1* significantly reduced *D. armandi*’s tolerance to α-pinene and limonene. The results of this study suggest that *DaABCH* genes have distinct functional characteristics, with *DaABCH1* potentially playing a role in the process of adapting to the host’s chemical defense. Findings from this study may provide a theoretical basis for the development of novel pest management strategies to effectively control this impactful pest. However, this study only preliminarily conducted functional experiments of *DaABCH* genes; the next step will involve metabolic validation, protein-level analysis and CRISPR/Cas knock-out experiments to provide more definitive evidence.

## Figures and Tables

**Table 1:** Sequence characteristics of *ABCH* genes and their deduced amino acids in *Dendroctonus armandi*.

Gene Name	GenBank Accession No.	ORF (bp)	Amino Acid Size	Topology	MW (KDa)	pI	Phospho-Rylation Sites (Ser/Thr/Tyr)	N-Glycosy-Lation Site	O-Glycosyl-Ation Site	Secondary Structure (α-Helix/β-Turn/Extended Strand/Random Coil)/%
*DaABCH1*	PV659137	2334	777	NBD-7TM	85.94	7.22	35/18/5	9	13	40.22/5.20/29.87/24.71
*DaABCH2*	PV659138	2133	710	NBD-5TM	79.36	7.81	32/24/7	6	2	40.82/5.42/28.34/24.42
*DaABCH3*	PV659139	2286	761	NBD-5TM	84.43	6.12	45/23/7	4	12	39.57/6.56/26.97/26.90

## Data Availability

The original contributions presented in this study are included in the article/Supplementary Materials. Further inquiries can be directed to the corresponding author.

## Supplementary Materials

The following supporting information can be downloaded at: https://www.mdpi.com/article/10.3390/insects16111075/s1. Table S1. Primer sequences used in the research. Figure S1. The survival rates of emerged adults (sex separated) in *D. armandi* after dsRNA injection at 48 h. Different letters indicate significant differences at *p* < 0.05. Post-hoc Tukey tests following one-way analysis of variance (ANOVA).
