# Supplementary material for: Transcriptional Response of ABCH Transporter Genes to Host Allelochemicals in Dendroctonus armandi and Their Functional Analysis
Source: Insects. 2025 Oct 22;16(11):1075. doi: 10.3390/insects16111075 (PMC12653539; doi:10.3390/insects16111075)
Supplement: Supplementary file 1 [file insects-16-01075-s001.zip › insects-3878323-supplementary.pdf]

Supplementary Material

Table S1 Primer sequences used in the research

Gene name	Sequence (5′ → 3′) Forward	Reverse	Purpose
DaABCH1	TTACGGACCGAACCCTAACC	CTTCTTCAAAGCCGCACGAC	cDNA
	Inner GTGGCGAAATGTGGGCGTGA	Inner ACGGCTTGCTGCCTGCGGTT	3' and 5'
	Outer ACCTGAGCGGTCGGTATGTG	Outer AACACCCACCAAGTCGGGGC	RACE
	AGTACATGGGAGAGCGCATG	GGCCTCGTTTAACTTTAAGG	Full-length
	GGTCGCTCTAACATCTTCCG	TCGCCTTTGCATTCCACTTC	qPCR
	**taatacgactcactataggg**CTTTATAGCGCTGGGG AAGG	**taatacgactcactataggg**ACTGGGTCACC ACATGGGAG	RNAi
DaABCH2	GAGTCGGTGGGGATGTTAAA	CGATAGGCATTGCTTCTGTG	cDNA
	Inner GCCTTCCCGGTACTCTGGCG	Inner GCGGAACGGGATCGGCGTAC	3' and 5'
	Outer GCGGAACGGGATCGGCGTAC	Outer GGTGTCAGTGTGAAACAACG	RACE
	CGTTTCACGATCATTTTCAA	CCTAACATTAAGGTCCGTCC	Full-length
	TGTGCGGCTTGGAAGTCTTC	TCCTTTCGGTCAGTGGGTAT	qPCR
	**taatacgactcactataggg**GCACGGTTCTTGGTG TTTTG	**taatacgactcactataggg**TCCTTTCGGTCA GTGGGTAT	RNAi
DaABCH3	GTAGAAACGATACCCCCAGA	GTGCCACAGCCATAAAGAAG	cDNA
	Inner GTTGTTTCACCCGTCTGCGA	Inner CGTCGTCCAGAATCGCTCCT	3' and 5'
	Outer ACCCATAGTGATGTTGTGTG	Outer CAATCAAATGTGCTTGCCGA	RACE
	TTTCAGAAATTTTGACGACA	CTAAACTTGAGCACCAGTAT	Full-length
	TATGGGTGTTGGGAGGACAG	GGCAGCATCAGCAAATTTAC	qPCR
	**taatacgactcactataggg**TAATGGAAAGGAATG AAGGA	**taatacgactcactataggg**TAATGGTAGGCA AGTGGAAG	RNAi
GFP	**taatacgactcactataggg**ATGGTGTTCAATGCTT TTCA	**taatacgactcactataggg**CTCTCTTTTCGT TGGGGTCT	RNAi

Note: T7 promoter sequences in the dsRNA synthesis primers were indicated in lowercase letters.

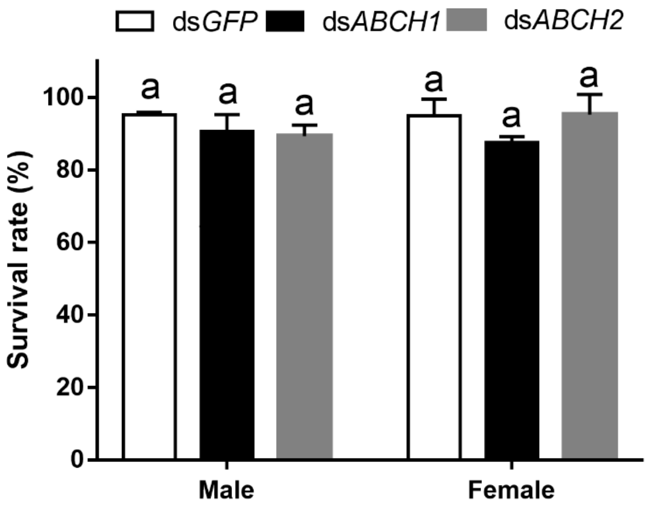

Figure S1. The survival rates of emerged adults (sex separated) in *D. armandi* after dsRNA injection at 48 h. Different letters indicate significant differences at $p < 0.05$. Post-hoc Tukey tests following one-way analysis of variance (ANOVA).
